# Surveillance for West Nile Virus in Clinic-admitted Raptors, Colorado

**DOI:** 10.3201/eid1302.051626

**Published:** 2007-02

**Authors:** Nicole Nemeth, Gail Kratz, Eric Edwards, Judy Scherpelz, Richard Bowen, Nicholas Komar

**Affiliations:** * Colorado State University, Fort Collins, Colorado, USA; † Centers for Disease Control and Prevention, Fort Collins, Colorado, USA; ‡ Rocky Mountain Raptor Program, Fort Collins, Colorado, USA

**Keywords:** West Nile virus, surveillance, raptors, Colorado, dispatch

## Abstract

In 2005, 13.5% of clinic-admitted raptors in northern Colorado tested positive for West Nile virus (WNV). Clinic-admitted–raptor surveillance detected WNV activity nearly 14 weeks earlier than other surveillance systems. WNV surveillance using live raptor admissions to rehabilitation clinics may offer a novel surveillance method and should be considered along with other techniques already in use.

West Nile virus (WNV; genus *Flavivirus*; family Flaviviridae) is an emerging pathogen of public health and veterinary importance. In North America, WNV has been associated with death in >198 species of birds, including >33 species of raptors (*1*). Many hawk and owl species are known to survive WNV infection (*2*–*5*). Presumably most raptors become infected from mosquito bites; however, some evidence suggests that infection may occur after consumption of infected prey items (*2*,*4*–*6*). Thus, raptors may be infected at a greater rate than nonraptors. Dead raptors and other birds (particularly corvids) have been used for early detection of WNV activity (*7*). However, once WNV activity is established in a location, birds that are highly susceptible to fatal infection are removed from the environment, and as a result, avian death rates should diminish (*8*). Raptors infected with WNV that are admitted to rehabilitation facilities, either because of WNV-associated illness or injury or for other unrelated complications, may serve as an alternate source for early detection of WNV infection.

## The Study

From 2002 through 2005, raptors originating in Colorado were bled by ulnar venipuncture and orally swabbed upon admission to the Rocky Mountain Raptor Program of Colorado State University. WNV was first detected in Colorado in August 2002, and testing of raptors was initiated in September (oral swabs) and October (serum samples). In all other years, samples were collected from early to late April through mid to late October. Specimens were tested for WNV-neutralizing antibodies by plaque-reduction neutralization test (PRNT) and for virus isolation by Vero cell plaque assay (*9*) or WNV antigen by VecTest WNV Antigen Detection Assay (Medical Analysis Systems, Ventura, CA, USA). Isolated viruses were identified as WNV by VecTest. To confirm that antibody-positive adult raptors were recently infected, we evaluated 90% neutralization titers in acute-phase and convalescent-phase serum samples collected ≈3 weeks apart. A 4-fold increase in titer was considered evidence of a recent infection. Cross-reactivity for another closely related North American flavivirus, Saint Louis encephalitis virus, was ruled out by comparing 90% neutralization titers. A 4-fold greater titer for 1 of the viruses indicated that particular virus as the etiologic agent for the infection. Utility of WNV detection in raptors was evaluated in relation to other existing WNV surveillance techniques in northern Colorado.

We report results from 323 raptors sampled from 2002 through 2005. Most of these (83%) originated from Weld and Larimer counties, which represent an area of 6,639 square miles, larger than Connecticut and Rhode Island combined. During the study, 38 raptors (11.8%) tested positive for WNV. Some were positive by both oral swab and seroconversion, while others were positive according to only 1 of these.. Usually, birds that were positive by oral swab only died before 1 or both blood samples could be collected, so we were unable to test for seroconversion.

In 2002, 17 raptors were tested (blood by PRNT and oral swab by plaque assay), 4 of which were seropositive for WNV between October 7 and November 15. In 2003, 52 birds were tested (serum by PRNT and oral swab by VecTest), 7 of which seroconverted and 5 of which were oral swab–positive. Positive samples were detected between July 17 and September 1. In 2004, 113 birds were tested by plaque assay of oral swab (no blood test), and 3 were found to be positive between July 28 and September 17. In 2005, 141 birds were tested (serum by PRNT and oral swab by plaque assay), of which 19 were positive (8 by seroconversion, 6 by virus isolation from swab, and 5 by both methods; Table). Positive results were from birds admitted between April 8 and September 21.

**Table:** Results of West Nile virus testing in Colorado raptors admitted to a rehabilitation clinic, April 1–October 15, 2005

Species	No. tested	No. positive (%)	No. seroconverted	No. swab positive	Earliest date of collection
Swainson hawk	28	8 (28.6)	5	3	Jun 27
Red-tailed hawk	13	4 (30.8)	2	3	Jul 28
Ferruginous hawk	1	1 (100.0)	0	1	Jul 31
American kestrel*	32	0	–	–	–
Peregrine falcon	2	1 (50.0)	1	1	Aug 16
Golden eagle	3	1 (33.3)	1	1	Aug 18
Great-horned owl	23	4 (17.4)	3	2	Apr 8
Common barn owl	24	0	–	–	–
Long-eared owl	5	0	–	–	–
Other species†	10	0	–	–	–
Total	141	19 (13.5)	12	11	Apr 8

*The proportion of positives for kestrel was significantly less than for all other species combined (p = 0.0046, α = 0.0056; Fisher exact test with Bonferroni adjustment for 9 comparisons). No other statistically significant associations were observed for the species tested.

†Includes burrowing owl (n = 2), sharp-shinned hawk (n = 2), Cooper's hawk (n = 1), eastern screech owl (n = 1), merlin (n = 1), osprey (n = 1), prairie falcon (n = 1), and saw-whet owl (n = 1).

To compare our test results with those from other surveillance systems for WNV, we limited our data to specimens collected April 1–October 15, 2005, from raptors originating in Weld or Larimer counties. In comparing the earliest date of detection for each of the surveillance methods in place in these counties, clinic-admitted raptor surveillance provided the earliest evidence of WNV activity (April 8), preceding all other WNV surveillance systems’ initial detections of WNV activity by nearly 14 weeks (Figure).

**Figure:**
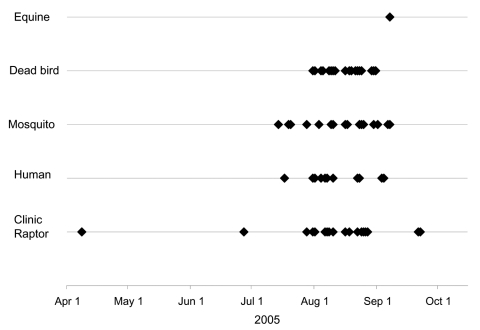
Chronology of detections of West Nile virus by various surveillance systems in place in Larimer and Weld Counties, northern Colorado, 2005. Confirmed human cases, mosquito, dead bird, and equine surveillance information provided by the Centers for Disease Control and Prevention’s ArboNet Surveillance System through October 15, 2005.

## Conclusions

The early detection of WNV in clinic-admitted raptors compared with other detections by surveillance systems in northern Colorado during 2005 points to the potential utility of raptor rehabilitation centers for WNV surveillance. Although other active surveillance systems require significant allocations of human resources, clinic-admitted raptor surveillance is a passive system that takes advantage of existing resources outside the traditional public health infrastructure. Nationwide, about 1,000 wildlife rehabilitation facilities admit ≈10,000 birds annually (P. Redig, pers. comm.). Participation in surveillance efforts provides rehabilitators with valuable diagnostic information and can be accomplished at no cost to the rehabilitator, provided that provisions are supplied.

The detection of WNV in an oral swab of a great horned owl in early April in Colorado was quite unexpected because of the early date. This bird was an uninjured nestling that was brought to the clinic for nurturing until it could be replaced into its original nest. The oral swab yielded a low number of infectious virus particles (2.5 PFU), and the nestling failed to develop clinical signs and failed to seroconvert. We believe that the oral cavity may have been contaminated by a recent prey meal provided by the bird’s parents shortly before admission. Although early spring transmission of WNV by mosquitoes to either the owlet or a prey animal is possible, persistent infection of the prey item is an alternative explanation. Experimentally infected hamsters develop chronically infected kidneys (*10*), and birds may also maintain persistent visceral infections (*2*).

If the early detection in the owl was an anomaly, the next earliest evidence of WNV activity from clinic-admitted raptors was June 28, which also preceded all other detections. The first confirmed human case of West Nile fever in the study area developed symptoms on July 17, and the first confirmed case of West Nile neurologic disease occurred on August 6 (Figure).

Although we have shown that a combination of serologic and oral swab testing increases the sensitivity of clinic-admitted raptor surveillance almost 2-fold, serolog testing has 3 important limitations: 1) blood sampling requires special training and expertise; 2) evidence of seroconversion requires 2 samples spaced apart by at least 2 weeks, and therefore reporting of positive results is significantly delayed by several weeks after onset of infection; and 3) neutralization tests can be prohibitively expensive and require extensive training, time, supplies, Biosafety Level-3 (BSL-3) lab facilities, and expertise in interpreting results, which are complicated by cross-reactions with closely related viruses. Limiting sampling to oral swabs reduces sensitivity; however, the savings in time and cost would permit a greater number of samples to be collected and tested. Although we used plaque assay for detecting WNV in oral swabs, which also requires BSL-3 laboratory facilities, our samples could have been tested with high sensitivity and specificity for WNV-specific RNA sequences by using reverse transcription–PCR, which requires a lower level of biosafety (*11*).

In conclusion, limited data from 1 small region of North America suggest that WNV surveillance using live raptor admissions to rehabilitation facilities should be considered along with other established surveillance methods already in use (*12*,*13*). Clinic-admitted raptors are most useful for early detection or continued detection of WNV activity. However, this form of surveillance is inadequate for quantifying local transmission risk.
